# Do Isopropylammonium Glyphosate and LiCl Impact the Spore Diversity and Functions of Aquatic Fungi Involved in Plant Litter Decomposition in Streams?

**DOI:** 10.3390/jox15030065

**Published:** 2025-05-01

**Authors:** Jorge Rodrigues, Hernâni Gerós, Manuela Côrte-Real, Fernanda Cássio

**Affiliations:** 1 CBMA—Centre of Molecular and Environmental Biology, University of Minho, Campus of Gualtar, 4710-057 Braga, Portugal; jorgerodrigues@bio.uminho.pt (J.R.); geros@bio.uminho.pt (H.G.); mcortereal@bio.uminho.pt (M.C.-R.); 2 IB-S—Institute of Science and Innovation for Bio-Sustainability, University of Minho, Campus of Gualtar, 4710-057 Braga, Portugal

**Keywords:** aquatic fungi, glyphosate-based herbicide, LiCl, fungal activity, fungal spore diversity, plant litter decomposition

## Abstract

Glyphosate based-herbicides are stressors of great concern because they can impact aquatic ecosystems. Similarly, lithium, a metal, is currently of concern because of its increasing use worldwide. Because glyphosate-based herbicides and lithium might co-occur in aquatic environments, there is a need to assess their impacts on aquatic organisms, such as aquatic fungi, as they play a key role in plant litter decomposition in streams. Microcosm assays were used to examine the effects of lithium and the herbicide isopropylammonium glyphosate (IPAG), alone or in mixtures, on microbial leaf mass loss, total fungal sporulation and biomass production. IPAG (alone and combined with LiCl) neither affected plant litter decomposition nor fungal biomass production, but boosted total fungal sporulation. *Dimorphospora foliicola*, the most tolerant species among the twelfth leaf inhabitant fungal species, is the major contributor to total fungal sporulation. IPAG interacts with LiCl in the total fungal sporulation and sporulation of *D. foliicola*, *A. tetracladia*, and *F. curvula*, indicating a species dependent-effect. IPAG alone or combined with LiCl greatly decreased the diversity of spores, as did as LiCl alone, but to a lesser extent. Finally, aquatic fungal communities reveal redundancy and resiliency to IPAG and LiCL, maintaining the health of aquatic ecosystems.

## 1. Introduction

Chemical pollution by pesticides is one of the main threats to freshwater ecosystems [1]. This is of special concern if it surpasses the frontiers of natural integrity because we may lose the ecological functions and services provided by natural ecosystems [2]. Limits for pesticide contamination, above which ecosystem integrity is compromised and the ecological services are likely lost forever, have not been determined yet [3]. Aquatic biota, including microorganisms, drives crucial ecological processes, such as organic matter turnover [4]. Plant litter decomposition is a process mediated by microorganisms, namely by bacteria and fungi, in which aquatic fungi play a major role in providing palatable resources for macroinvertebrate detritivores. This process drives the flux of carbon and energy across the entire food chain [5]. Aquatic fungal communities and the processes they drive have been used for studying the effects of stressors (e.g., microplastics, [6]. Indeed, due to the rapid reproduction rate and rapid adaptation to stressors, those microbial communities may forecast and allow extrapolating the consequences of pesticide contamination for stream ecological functions [3,7].

Pesticides comprising insecticides, herbicides, and fungicides are a group of potentially toxic substances that are capable of disrupting the microbial community structure and function in aquatic habitats (for a revision, see [8]). They may reach freshwater habitats through several pathways, including spray drift and runoff [9,10]. Indeed, field studies in streams near agricultural sites or vineyards have shown that the actual concentrations of a variety of pesticides affect aquatic fungi and lead to the reduction of plant litter decomposition [11]. Agriculture in large scale mostly depends on the use of pesticides and the herbicide glyphosate (N-(phosphonomethyl)glycine) stands out as one of the most used by farmers, as it is highly effective and cost-efficient [12]. The 5-enolpyruvylshikimate-3-phosphate synthase (EPSPS) of the shikimate pathway is the target enzyme of glyphosate, thereby interrupting the biosynthesis of the aromatic amino acids, phenylalanine, tryptophan, and tyrosine, and inhibiting plant secondary metabolism [13]. Glyphosate also damages cellular structures and disrupts chloroplasts, membranes, and cell walls, reduces chlorophyl content, and compromises nucleic acid synthesis, photosynthesis, and respiration [4]. The massive application of glyphosate-based herbicides results in the contamination of streams by glyphosate. Glyphosate occurrence near cultivated areas was reported to be between 0.42 to 2.93 mgL^−1^ [14,15]. In the worst-case scenarios, predicting an accidental spill or direct overspray in water, the glyphosate concentration can reach 7.12 to 24.9 mgL^−1^ [16,17,18,19]. As commercial formulations of glyphosate are mixtures of the active ingredient, the concentrations applied to the environment, and, in particular, in the case of isopropylammonium glyphosate (IPAG) used in the present study, would have been 1.43-fold higher than that of the active ingredient.

Lithium is abundant in the Earth’s crust, reaching soils and water bodies by weathering processes, contaminating them and affecting living organisms [20]. In addition, discharges of industrial effluents from smelting and mining operations [21] and inadequate disposal of products that use lithium in their manufacture (e.g., batteries, detectors, or atomic reactors) [22] contribute to environmental contamination. As such, worldwide, lithium contamination is increasing and imposing serious threats to the environment and humans [23]. In particular, certain regions of Portugal possess abundant lithium, and prospecting activities are set to begin, raising concerns about the anticipated increase in environmental contamination with lithium in the near future. The lithium concentration in water bodies depends on geology, topography, and hydrogeology, along with other variables [24]. Lithium concentrations in surface waters, namely rivers and ponds, can range from 1 to a maximum threshold of 10 µgL^−1^. Indeed, lithium has been found at concentrations ranging from 20 to 91 µgL^−1^ in surface waters and between 0.02 and 97 µgL^−1^ in ground waters of the South East of Ireland [25], though it can reach concentrations up to 500 µgL^−1^ ([26] reviewed in [27]). As far as we know, there is no established regulatory maximum admissible concentration for lithium in surface waters. In particular, different countries around the world do not have maximum permissible limits or guideline recommendations for lithium concentrations in drinking water ([28] reviewed in [29]). One of the exceptions is in the United States, where the EPA has set 40 µgL^−1^ of lithium as a regional screening level for residential tap water (reviewed in [29]).

The rising demand for renewable energy systems and more efficient technologies has significantly increased the demand for lithium-ion batteries. To meet this need, battery production has scaled up, resulting in a growing volume of spent batteries. As this waste stream expands, effective management becomes increasingly important. Unfortunately, there are still no universal or standardized disposal strategies in place. Improper handling and disposal will lead, in the near future, to environmental pollution, especially the contamination of water sources, which can disrupt entire ecosystems and harm multiple trophic levels [27]. Although the co-occurrence of glyphosate and lithium has not been documented in streams, their simultaneous presence is plausible, particularly because of their increased release into the environment.

As many aquatic habitats are embedded within agricultural landscapes, they may unintentionally be exposed to agricultural pesticides, which may affect organisms, including aquatic fungal decomposers [10]. However, despite the fundamental role of microorganisms in aquatic food webs, our understanding of how their activity and diversity are affected by the exposure to pesticides and lithium is quite limited [22,29]. Here, we aimed to clarify if IPAG and lithium, alone or in mixtures, might affect fungal fitness and plant litter decomposition under the hypotheses that (i) the exposure to IPAG and lithium can affect microbial activity, namely leaf litter decomposition, fungal sporulation, and fungal biomass production; and (ii) IPAG and lithium may interact with each other in mixtures. We used microcosm assays composed by oak leaves colonized by stream-dwelling aquatic microbes and monitored plant litter decomposition, fungal sporulation, and fungal biomass production after exposure to IPAG and LiCl alone and in combination at environmentally realistic concentrations. The novelty of our study is to assess the effects of glyphosate and lithium alone and in mixtures in a relevant ecological process, the plant litter decomposition, and, in particular, on spore diversity and functions of aquatic fungi driving the process. This can be particularly useful for managers of ecosystems to maintain biodiversity and ecological functions.

## 2. Methods

### 2.1. Leaf Processing, Study Area, and Field Procedures

Senescent oak leaves were collected (in October/November 2016), dried at room temperature, and stored in a dry atmosphere. About 2 years later (January 2019), the leaves were immersed in deionized water to soften for 24 h, cut into discs of 12 mm diameter, and then placed inside fine mesh bags (0.5 mm, 16 × 20 cm size) to prevent macroinvertebrate colonization. The bags were immersed (1 February 2019) for 12 days in the Oliveira stream, Northwest Portugal, for microbial colonization. The stream water physicochemical parameters from where microbial communities were retrieved were measured in situ using field probes (Multiline F/set 3 no. 400327 (WTW) British-American multinational company, Washington, DC, USA). After 12 days, colonized leaf discs from each bag were rinsed with deionized water to remove sediments and adhering invertebrates before carrying out the microcosm assays.

### 2.2. Microcosm Assays

Leaf discs were placed in plastic Erlenmeyer flasks containing 100 mL of a sterilized commercial water (Fastio, Chamoim, Portugal) with IPAG and/or LiCl under shaking at 17 °C for 49 days. Mineral water was used instead of stream water to ensure a controlled medium chemical composition. To determine ergosterol and leaf mass loss, 6 and 70 leaf discs were used per replicate, respectively, with 3 replicates for each condition. We tested five concentrations of IPAG (0.1, 2.5, 50, 500, 1500 µgL^−1^; Genyen, Loural, Portugal), one of the commercial formulation of the herbicide-based Sereno, and three concentrations of LiCl (0.1, 5, 100 µgL^−1^; Sigma Aldrich, St. Louis and Burlington, MA, USA), alone and in all possible combinations, with 3 replicates per treatment in a total of 72 microcosm assays. Control microcosm assays contained microbial colonized leaf discs without chemicals. IPAG and LiCl concentrations chosen were within the range reported in the literature in environmental samples. As glyphosate can be degraded, the medium (mineral water with IPAG and/or LiCl) used in the microcosm assays was renewed every week by decantation and subsequently addition of new water and toxicants at the desired concentrations according to other studies [6] to ensure continued exposure to the selected toxicant concentrations. Every week, 40 mL of the decanted medium from each treatment was stored in glass bottles for spore identification and counting. The spore suspensions were preserved by adding 800 µL of formaldehyde and 40 µL of Triton X (Sigma-Aldrich) 15% (*w*/*v*) to prevent their germination and adherence to the bottles. Leaf discs for assessment of mass leaf loss and fungal biomass were frozen at −20 °C. The leaf discs were lyophilized (Bioblock Scientific Christ Alpha 2-4 LD Plus) and kept in a desiccator until further use.

### 2.3. Leaf Mass Loss

Leaf mass loss was estimated by weighing, with an accuracy of 0.01 g (Mettler Toledo AG245), a subset of lyophilized leaf discs before and after the microcosm assay.

### 2.4. Total Fungal Sporulation and Fungal Diversity

A suspension of spores from each treatment was used to identify and count fungal spores. Spore suspensions were filtered through 0.45 µm filters and stained with 0.1% cotton blue in lactic acid. Then, the filters were scanned under a light microscope for spore identification and counting, at a magnification of 400× (light microscope; Leica Biomed, Heerbrugg, Switzerland).

### 2.5. Fungal Biomass

Fungal biomass was based on ergosterol content on leaves according to [30]. Five lyophilized leaf discs were refluxed in 10 mL of 0.8% KOH-methanol for 30 min at 80 °C. Then, the resulting lipid extract was purified by solid-phase extraction followed by high-performance liquid chromatography (HPLC; Beckman Golden System, Beckman Coulter, Inc., Brea, CA, USA), using a Lichrospher RP-18 column (25 by 0.40 cm; Merck, Darmstadt, Germany). The system was run isocratically with HPLC-grade methanol as the mobile phase (33 °C; 1.4 mL min^−1^). Ergosterol was detected at 282 nm and quantified based on a standard curve of ergosterol (Sigma, Kanagawa, Japan) in isopropanol.

### 2.6. Data Treatment

The effects of increasing concentrations of IPAG and LiCl on fungal biomass, sporulation rates, and leaf mass loss were tested by ANOVAs. Two-way ANOVAs were used to compare the main effects of the independent variables, IPAG and LiCl, in comparison with the control. Bonferroni post hoc correction was only performed when the two independent variables interacted, and was used to assess differences among groups (IPAG and LiCl). Data were previously checked for normal distribution (Shapiro test) and homoscedasticity (Bartlett test). When the assumptions of ANOVA tests were not met, data were transformed to natural logarithms. When this transformation was not possible (values below 1), the Kruskal–Wallis non-parametric test was performed instead of ANOVA. In this case, if significant, multiple comparisons were performed with an equivalent test to the Tukey test.

All statistics were performed in R, and all graphics were performed in GraphPad Prism Software version 8.0.1.

## 3. Results

To study the effects of isopropylammonium glyphosate (IPAG) and LiCl, alone and in combination, on microbial decomposition of plant litter and on the aquatic fungal parameters, namely total sporulation and spore diversity as well ergosterol content, microcosm assays were carried out with oak leaves previously colonized by aquatic microbes in the Oliveira stream. The physicochemical parameters of the stream indicated a moderate acidity (pH 6.86), temperature of 9.9 °C, high dissolved oxygen concentration (86.3%), and low conductivity (32 µScm^−1^). The results obtained are described in the following sections.

### 3.1. Effects of Isopropylammonium Glyphosate and LiCl Alone and in Combination on Microbial Decomposition of Plant Litter and Total Sporulation and Spore Diversity of Aquatic Fungi

**Figure 1:**
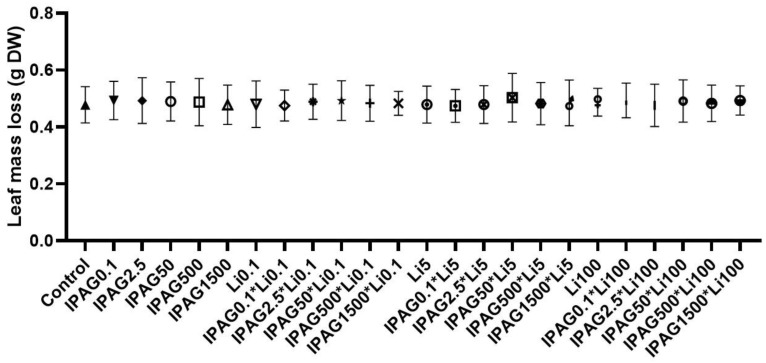
Effects of isopropylammonium glyphosate (IPAG) 1500 µgL^−1^ or LiCl (Li) alone (0.1 or 100 µgL^−1^) and in combination on plant litter decomposition by colonized aquatic microbes, assessed by loss of leaf mass (g of leaf mass in dry weight, DW) 49 days after non-exposure or exposure to both chemicals in microcosms assays. Control refers to loss of leaf mass in the absence of IPAG and Li. In the XX axis, * means combination of IPAG and LiCl. Data presented are the mean values of three replicates and the respective standard deviations.

As noted in the introduction, the concentrations of glyphosate most often found in the environment (0.42 to 2.93 mgL^−1^, [15]) correspond to concentrations of isopropylammonium glyphosate (IPAG) 1.43-fold higher (0.57 to 3.96 mgL^−1^). Also, as noted in the introduction, the environmental lithium concentrations reported in the literature range from 0.02 to 97 µgL^−1^ [25,26,27]. In the present study, the effects of IPAG and LiCl, alone or in combination, on plant litter decomposition by aquatic microbes was assessed by the loss of leaf mass in dry weight. For this purpose, 0.1, 2.5, 50, 500, and 1500 µgL^−1^ of IPAG and 0.1, 5, and 100 µgL^−1^ of LiCl were used, alone or in combination. All the concentrations of IPAG and LiCl tested, as well of their combinations, did not affect the leaf mass loss (*p* > 0.05, Figure 1).

**Figure 2:**
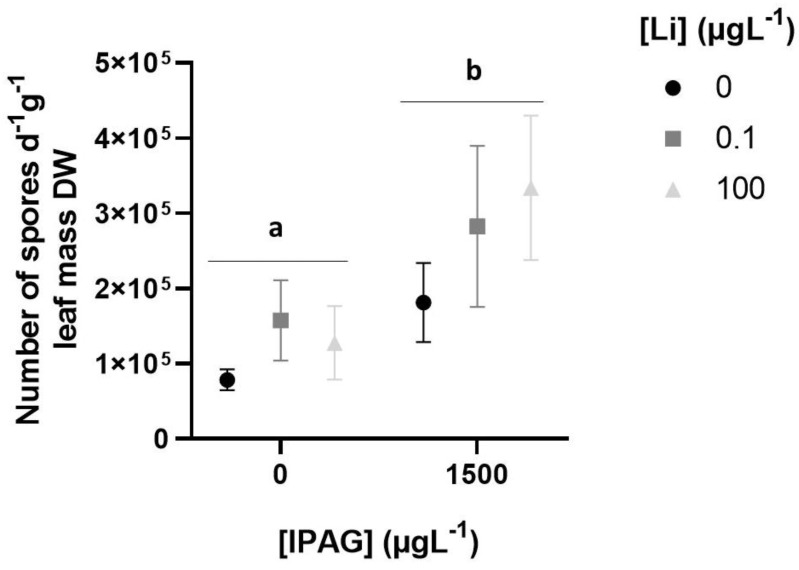
Effects of isopropylammonium glyphosate (IPAG) and LiCl (Li) alone and in combination on the total sporulation of aquatic fungi, expressed as the total number of spores per day and per gram of leaf mass in dry weight (DW). Samples treated with IPAG 1500 µgL^−1^, Li 0.1 µgL^−1^ or Li 100 µgL^−1^, or both with IPAG and Li (in grey colors), were compared with non-treated samples used as controls (in black). Data presented are the mean values of three replicates and the respective standard deviations. Significant statistical differences are indicated with small caps (a,b) (*p* < 0.05).

Total fungal sporulation rate, assessed by estimation of the number of spores per day and per gram of leaf mass, increased significantly 49 days after exposure to IPAG (two-way ANOVA, F = 29.570, *p* = 0.000151) and LiCl (two-way ANOVA, f = 6.086, *p* = 0.014971). There was no interaction between IPAG and LiCl (two-way ANOVA, F = 0.650, *p* = 0.539619). However, Bonferroni post hoc tests revealed that the adjusted *p* values were significantly different when comparing the concentrations of IPAG (0 and 1500 µgL^−1^) (*p* = 0.00054), while there were no significant differences between LiCl (0 and 0.1 and 100 µgL^−1^) (*p* > 0.05) and between IPAG alone and in combination with LiCl (0.1 and 100 µgL^−1^) (*p* > 0.05) (Figure 2).

**Figure 3:**
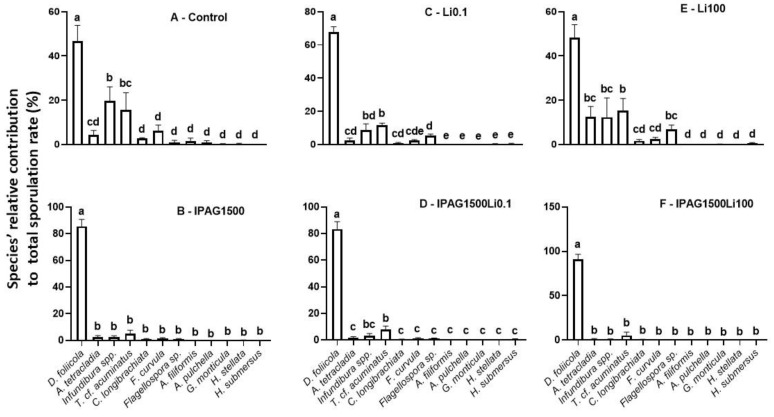
Effects of isopropylammonium glyphosate (IPAG) and LiCl (Li) alone and in combination on aquatic fungal spore diversity through the assessment of the relative contribution of each species to the total sporulation rate. Relative contribution (%) of each aquatic fungal species to total sporulation rate in samples: (**A**), untreated (Control); (**B**), exposed to IPAG 1500 µgL^−1^; (**C**), Li 0.1 µgL^−1^; (**D**), IPAG 1500 µgL^−1^ and Li 0.1 µgL^−1^; (**E**), exposed to Li 100 µgL^−1^; (**F**), exposed to IPAG 1500 µgL^−1^ and Li 100 µgL^−1^. Data presented are the mean values of three replicates and the respective standard deviations. Significant differences of the relative contribution of each species to the total sporulation are indicated by small caps (*p* < 0.05).

Based on fungal spore morphology, the relative sporulation rates (%) of 12 fungal species (*Dimorphospora foliicola*, *Infundibura* spp., *Triscelophorus* cf. *acuminatus*, *Flagellospora curvula*, *Articulospora tetracladia*, *Clavatospora longibrachiata*, *Flagellospora* spp., *Anguillospora filiformis*, *Alatospora pulchella*, *Goniopila monticula*, *Heliscella stellata*, and *Heliscus submersus*) identified on decomposing leaves 49 days after non-exposure and exposure to IPAG and Li, alone or in combination, were estimated. The species *D. foliicola*, *Infundibura* spp., *T.* cf. *acuminatus*, *F. curvula*, *A. tetracladia*, and *C. longibrachiata* were the six most abundant, as they showed the highest relative sporulation rates. The abundance of *D. foliicola* was significantly higher than that of other species (*p* < 0.05, Figure 3A). Infundibura spp. was the second most predominant species, with a sporulation similar to that of *T.* cf. *acuminatus* (Bonferroni post hoc test, adjusted *p* = 1; Figure 3A). Both species had significantly lower sporulation than *D. foliicola* (Bonferroni post hoc test, adjusted *p* = 3.0 × 10^−7^ and adjusted *p* = 1.9 × 10^−8^, respectively (Figure 3A)).

IPAG (1500 µgL^−1^) significantly increased the relative contribution of *D. foliicola* to the total sporulation (Kruskal–Wallis chi-squared = 33.154, df = 11, *p* = 0.0004968; Figure 3B). All the other species had similar sporulation rates (Bonferroni post hoc test, adjusted *p* > 0.05), with IPAG (1500 µgL^−1^) significantly decreasing the relative contribution of *A. tetracladia*, *F. curvula*, *T.* cf. *acuminatus*, *C. longibrachiata*, and *Infundibura* spp. (Figure 3B). In the treatment with LiCl 0.1 µgL^−1^, *D. foliicola* was dominant (Bonferroni post hoc test, adjusted *p* = 2 × 10^−16^). *T.* cf. *acuminatus* followed *D. foliicola* as the second most abundant species, similarly to *Infundibura* spp. (Bonferroni post hoc test, adjusted *p* = 1) and *Flagellospora* spp. (Bonferroni post hoc test, adjusted *p* = 1), but had a higher sporulation rate than all the other species (Bonferroni post hoc test, adjusted *p* > 0.05; Figure 3C). In the treatment with LiCl 100 µgL^−1^, the contributions of more species increased and equaled the contribution of *T.* cf. *acuminatus* and *Infundibura* spp. (Figure 3E). *D. foliicola* remained as the higher contributor to total sporulation (Bonferroni post hoc test, adjusted *p* < 0.05), followed by *T.* cf. *acuminatus* with a contribution similar to that of *Infundibura* spp., *A. tetracladia*, and *Flagellospora* spp. (Bonferroni post hoc test, adjusted *p* > 0.05). The relative sporulation of *Infundibura* spp., *A. tetracladia*, and *Flaggellospora* spp. was not different from that of *C. longibrachiata* and *F. curvula* (Bonferroni post hoc test, adjusted *p* > 0.05). LiCl 100 µgL^−1^ was the treatment that led to a higher number of species contributing to the total sporulation (Figure 3E).

When IPAG 1500 µgL^−1^ was combined with LiCl 0.1 or 100 µgL^−1^, the contribution of *D. foliicola* to the total sporulation was similar to that with IPAG 1500 µgL^−1^ (Bonferroni post hoc test, adjusted *p* = 1), and so, higher than the control (Bonferroni post hoc test, adjusted *p* = 9.1 × 10^−7^; Figure 3A,B,D,F). Under these conditions IPAG impacted the effect of LiCl 0.1 and 100 µgL^−1^, reducing the number of species contributing to sporulation, which indicates an interaction between IPAG and LiCl (two-way ANOVA, F = 10.420, *p* = 0.00238). In the treatments with IPAG 1500 µgL^−1^ without and with LiCl 0.1 and 100 µgL^−1^ (Figure 3B,D,F), there was mainly no clear difference between *Infundibura* spp. and *T.* cf. *acuminatus* and the other species, while in the treatments without IPAG (control and LiCl 0.1 µgL^−1^ and 100 µgL^−1^) (Figure 3A,C,E), the contribution of these two species to the total sporulation rate was more evident compared to *D. foliicola*.

**Figure 4:**
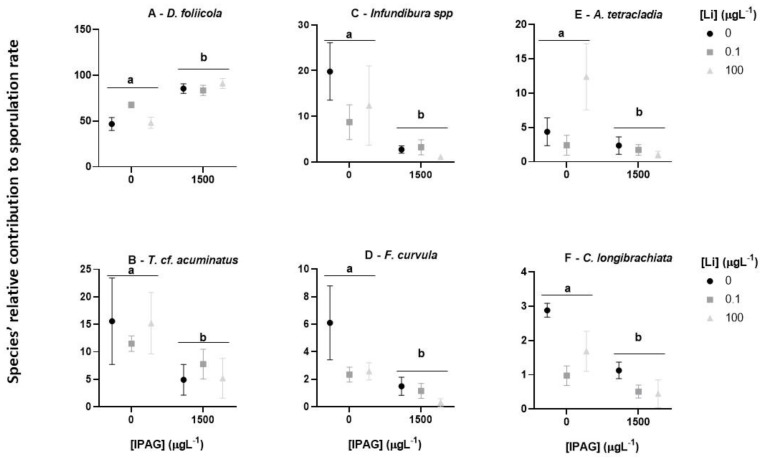
Effects of isopropylammonium glyphosate (IPAG) and LiCl (Li) alone and in combination on the relative sporulation rate (%) of each fungal species (**A**–**F**). Control, IPAG 1500 µgL^−1^, Li 0.1 µgL^−1^, Li 0.1 µgL^−1^ with IPAG 1500 µgL^−1^, Li 100 µgL^−1^, and Li 100 µgL^−1^ with IPAG 1500 µgL^−1^. Data presented are the mean values of three replicates and the respective standard deviations. Significant differences are indicated with small caps (a,b). (*p* < 0.05).

Although IPAG and Li had an interactive effect on the total fungal sporulation rate, whether it depended on the fungal species remained unknown. To address this question, the impact of the combined treatments with IPAG and LiCl on the relative contribution of each of the six most relevant fungal species was assessed and compared with the treatments with each toxicant alone (Figure 4).

The relative sporulation rates of *D. foliicola* for the different conditions tested were higher (above 50%) than those of *T.* cf. *acuminatus*, *Infundibura* spp., *A. tetracladia* (below 20%), *F. curvula*, and *C. longibrachiata* (below 6%). Moreover, the sporulation rates of *T.* cf. *acuminatus*, *Infundibura* spp., *F. curvula*, *A. tetracladia*, and *C. longibrachiata* were lower in the treatments with IPAG 1500 µgL^−1^ (with or without LiCl) than in the control and treatments with Li alone (Figure 4B–F).

*D. foliicola* data showed there was an interaction of IPAG and LiCl (two-way ANOVA, F = 10.420, *p* = 0.00238). The treatments with IPAG (with and without LiCl 0.1 and 100 µgL^−1^) were higher than the control (Bonferroni post hoc test, adjusted *p* = 9.1 × 10^−7^). The treatments with LiCl 0.1 and 100 µgL^−1^ (without IPAG) were equal to the control (Bonferroni post hoc test, adjusted *p* = 1), and LiCl 0.1 was equal to LiCl 100 µgL^−1^ (Bonferroni post hoc test, adjusted *p* = 1) (Figure 4A).

*T.* cf. *acuminatus* data showed there was no interaction of IPAG and LiCl (two-way ANOVA, F = 1.074, *p* = 0.37243). The treatments with IPAG (with and without LiCl 0.1 and 100 µgL^−1^) were lower than control (Bonferroni post hoc test, adjusted *p* = 0.00095). The treatments with LiCl 0.1 and 100 µgL^−1^ (without IPAG) were equal to control (Bonferroni post hoc test, adjusted *p* = 1; Figure 4B).

*Infundibura* spp. showed no interaction between IPAG and LiCl (two-way ANOVA, F = 2.869, *p* = 0.0959). The contribution of this species to total sporulation was low in treatments with IPAG (with or without LiCl) (Bonferroni post hoc test, adjusted *p* = 1.2 × 10^−5^) compared with control, but also, there were no differences between control and the treatments with LiCl (0.1 and 100 µgL^−1^, without IPAG), (Bonferroni post hoc test, adjusted *p* > 0.05; Figure 4C).

*F. curvula* data showed there was interaction of IPAG and LiCl (two-way ANOVA, F = 4.158, *p* = 0.04247). The treatments with IPAG 1500 µgL^−1^ (with or without LiCl 0.1 and 100 µgL^−1^) presented lower contribution to total sporulation than the treatments without IPAG (control, LiCl 0.1 and 100 µgL^−1^) (Bonferroni post hoc test, adjusted *p* = 0.0011). The treatments with LiCl 0.1 and 100 µgL^−1^, without IPAG, had equal effects compared to control (Bonferroni post hoc test, adjusted *p* > 0.05; Figure 4D).

*A. tetracladia* data showed that there was an interaction between IPAG and LiCl (two-way ANOVA, F = 8.821, *p* = 0.004401). IPAG 1500 µgL^−1^ decreased the sporulation of this species compared to the control (Bonferroni post hoc test, adjusted *p* = 0.0042) and in control and treatments with LiCl 0.1 and 100 µgL^−1^ without IPAG, the sporulation rates were similar (Bonferroni post-Hoc test, adjusted *p* > 0.05; Figure 4E).

*C. longibrachiata* showed no interaction of IPAG and LiCl (two-way ANOVA, F = 1.361, *p* = 0.293358). IPAG 1500 µgL^−1^ (with or without LiCl 0.1 and 100 µgL^−1^) decreased fungal sporulation (Bonferroni post hoc test, adjusted *p* = 0.0038), but the treatments of LiCl 0.1 and 100 µgL^−1^ (without IPAG) had no effects (Bonferroni post hoc test, adjusted *p* > 0.05; Figure 4F).

### 3.2. Effects of Isopropylammonium Glyphosate and LiCl Alone or in Combination on Fungal Biomass

**Figure 5:**
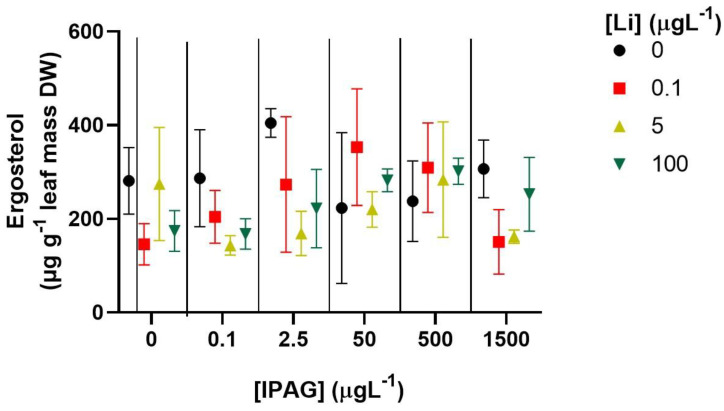
Effects of isopropylammonium glyphosate (IPAG) 0–1500 µgL^−1^ and LiCl (Li) 0.1–100 µgL^−1^ alone or in combination on the fungal biomass, assessed by the concentration of ergosterol on decomposing leaves, 49 days after incubation. Data presented are the mean values of three replicates and the respective standard deviations.

The effects of IPAG and LiCl, alone or in combination, on fungal biomass were assessed by ergosterol content of fungi associated with oak leaves. Effects of IPAG on ergosterol content were marginally significant (two-way ANOVA, F = 2.202, *p* = 0.0694), and the effect of LiCl was significant (two way-ANOVA, F = 3.012, *p* = 0.0391), and there was an interaction between the two factors (two Way-ANOVA, F = 2.260, *p* = 0.0167). However, no differences were observed regarding fungal biomass between the IPAG groups (Bonferroni post hoc test, adjusted *p* > 0.05) and the LiCl groups (Bonferroni post hoc test, adjusted *p* > 0.05). The only group that was marginally different from control was the LiCl 5 µgL^−1^ group (Bonferroni post hoc test, adjusted *p* = 0.089) (Figure 5).

## 4. Discussion

Though pesticides and metals are toxic to aquatic organisms that are relevant for nutrient cycling and therefore essential to life, the effects of the most commonly used pesticides have not been completely unveiled. We used a natural microbial community composed by bacteria and fungi to test the effects of isopropylammonium glyphosate (IPAG) and LiCl. We want to point out that aquatic fungi have the main role on plant litter decomposition in streams, making them an ideal microorganism to study the effects of these pesticides. Earlier studies reported that exposure to pesticides did not affect leaf microbial decomposition rates [31,32]. Accordingly, isopropylammonium glyphosate (IPAG) and LiCl had no effects on leaf litter decomposition by aquatic microbes. In contrast, treatments with IPAG alone or in combination with LiCl did change the total fungal sporulation significantly. The failure in sporulation does not necessarily translate in the absence of fungal taxa and non-contribution to plant litter decomposition [33,34]. This suggests that these parameters may not be related, and may explain why the treatment with IPAG alone or in combination with LiCl affects the total fungal sporulation, but not leaf microbial breakdown. As opposed to IPAG, treatments with LiCl alone did not change the total fungal sporulation significantly, but in the presence of IPAG, they did, which suggests an interaction between LiCl and IPAG. To ascertain the weight of each species in the total sporulation changes, and eventually in the number species contributing to it, the toxic effects of both IPAG and LiCl on the relative contribution of each of the six species most relevant to the total sporulation were assessed. The results revealed that the significant increase in total fungal sporulation (*p* < 0.05) upon treatment with IPAG alone and in combination LiCl, was mainly due to *D. foliicola*, the most abundant species found in both untreated and treated samples. Accordingly, *D. foliicola* was also found to be a predominant species in a polluted site of the Este River [35]. Altogether, these data support the notion that this species is tolerant to contaminants. However, in contrast to *D. foliicola*, *T.* cf. *acuminatus*, *Infundibura* spp., *F. curvula*, *A. tetracladia*, and *C*. *longibrachiata* decreased their relative contribution to the total sporulation. Moreover, the analysis of the effects of each treatment on the relative contribution of each species to the total sporulation showed a change in the number of the natural inhabitant fungal species, thus impacting the diversity of spores and function of the fungal community. Indeed, in samples treated with IPAG 1500 µgL^−1^, alone and in combination with LiCl (0.1 and 100 µgL^−1^), there was a decrease from six predominant species to two or one species (*D. foliicola* in treatments with IPAG 1500 combined with LiCl 0.1 µgL^−1^, and *D. foliicola* and *T.* cf. *acuminatus* in the combined treatment with IPAG 1500 µgL^−1^ and LiCl 100 µgL^−1^, respectively), while for the treatments with LiCl 0.1 or 100 µgL^−1^ alone, there was a decrease to four and five predominant species, respectively. However, in treatments with LiCl 0.1 and 100 µgL^−1^, *Flagellospora* spp. became predominant, replacing *A. tetracladia* and *F. curvula* in LiCl 0.1 µgL^−1^ and *F. curvula* in LiCl 100 µgL^−1^. Altogether, the combined treatments also showed that the effects of IPAG mainly overlapped the effects of LiCl 0.1 and 100 µgL^−1^, as IPAG combined with LiCl displayed the same reduction as IPAG alone in the number of predominant species. Since *D. foliicola* followed by *Infundibura* spp., *T.* cf. *acuminatus* and *A. tetracladia* were the most predominant species found in both untreated and treated samples, they are likely to be more tolerant to IPAG and LiCl alone and in combination than the other inhabitant species, indicating that the fungal response to pesticides is species-dependent. Some earlier evidence indicates that species with different levels of tolerance to contaminants may co-occur in streams, serving as a buffer against environmental changes [36]. Analysis of the effects of each treatment on the relative contribution of each species to the total sporulation revealed that with *D. foliicola*, *A. tetracladia*, and *F. curvula*, there was an interaction between IPAG and Li, while with *T.* cf. *accuminatus*, *Infundibura* spp., and *C. longibrachiata*, there was not. In accordance with leaf mass loss, fungal biomass is not affected by IPAG or LiCl alone or in combination. Glyphosate is co-metabolized as a carbon, nitrogen, and phosphorus source in microbiological cultures including bacteria, actinomycetes, fungi, and other non-identified microorganisms (for a revision, see [14]). However, to our knowledge, there are no data on degradation by aquatic fungi. This factor could explain the lack of effects on leaf mass loss and fungal biomass.

IPAG and LiCl alone and in combination did not affect fungal biomass. Other studies revealed that the use of structural and functional metrics might give uncoupled outputs, as the response in a metric may not be equal in another metric [37]. In our study, the effects of IPAG and LiCl alone and in combination on the structural metric (fungal sporulation) diverged from the functional metric (fungal biomass production), as the treatments that increased total fungal sporulation did not cause an increase in fungal biomass. Nonetheless, the observed negative effects on fungal sporulation might compromise the spread of fungi in natural ecosystems in the long term, but the absence of negative effects on fungal biomass should not lead to lower leaf mass decomposition and less suitable resources for higher trophic levels. These differential effects may be partially explained by the complexity of the ecosystem process studied.

## 5. Conclusions

The effects of pesticides on aquatic microbial diversity can lead to an impoverished fungal community, compromising its overall performance, unless redundant species can ensure functions. In the present study, changes in aquatic fungal community composition upon exposure to IPAG and LiCl were found. Unexpectedly, this may contribute to maintaining fungal functions under stressed conditions imposed by pesticides, such as IPAG and lithium alone or in combination, as to some extent, sensitive species are substituted by others more tolerant to stressors. Because different fungal species and the total fungal sporulation varied with treatments, we can conclude that shifts in community structure might have ensured the community function (leaf litter decomposition), probably because redundant species in the community could maintain the functions of those species that disappeared. Accordingly, several studies showed considerable redundancy between aquatic fungi during leaf litter decomposition. Both the aforementioned fungal structural and functional community responses may be a safeguard for the functional stability of fungal-mediated processes, namely plant litter decomposition and nutrient cycling in streams chronically affected by fungicides.

In summary, we conclude that aquatic fungal responses varied with the IPAG and LiCl concentrations as well as with their co-occurrence, which reinforces the need to assess the effect of mixtures in the environment. LiCl did not affect the studied fungal parameters. IPAG, alone and in combination with LiCl, had a high impact on fungal spore diversity as well as on the relative contribution of each fungal species to the process of plant litter decomposition, either increasing or decreasing their contribution to total sporulation, though it did not affect either fungal biomass or plant litter decomposition by aquatic microbes. Fungal aquatic communities, involved in the nutrient cycling important to higher trophic levels and ultimately to the quality of our lives, are at risk due to their exposure to environmental contaminants. The present study, together with the assessment of leaf mass loss and biomass formation, explored the fact that spores of aquatic fungi have specific morphological characteristics, which allowed us to identify the sporulating species through simple light microscopic observation. Nonetheless, the spores identified reflected the species that had colonized the plant litter earlier, but not the species that were actually present at the time of sampling. To this end, the use of molecular tools could be an enhanced approach, allowing one to record the history of the contaminants in litter samples. The present study offers novel insights into the resilience of aquatic fungal communities to glyphosate-based herbicides and lithium pollution, two increasingly prevalent environmental contaminants whose combined effects on freshwater ecosystems have been scarcely investigated. Finally, this study is important for ecotoxicologists, freshwater ecologists, and environmental policymakers seeking to understand the related ecological risks.

## Data Availability

The data presented in this study are openly available in Repositorium at https://repositorium.sdum.uminho.pt.

## References

[1] Dudgeon D. (2019). Multiple threats imperil freshwater biodiversity in the Anthropocene. Curr. Biol..

[2] Deeksha, Shukla A.K. (2022). Ecosystem Services: A Systematic Literature Review and Future Dimension in Freshwater Ecosystems. Appl. Sci..

[3] Schreiner V.C., Feckler A., Fernández D., Frisch K., Muñoz K., Szöcs E., Zubrod J.P., Bundschuh M., Rasmussen J., Kefford B. (2018). Similar recovery time of microbial functions from fungicide stress across biogeographical regions. Sci. Rep..

[4] Wang C., Lin X., Li L., Lin S. (2016). Differential growth responses of marine phytoplankton to herbicide glyphosate. PLoS ONE.

[5] Pascoal C., Cássio F., Marcotegui A., Sanz B., Gomes P. (2005). Role of fungi, bacteria and invertebrates in leaf litter breakdown in a polluted river. J. N. Am. Benthol. Soc..

[6] Trabulo J., Pradhan A., Pascoal C., Cássio F. (2022). Can microplastics from personal care products affect stream microbial decomposers in the presence of silver nanoparticles?. Sci. Total Environ..

[7] Graham E.B., Knelman J.E., Schindlbacher A., Sicilliano S., Breulmann M., Yannarell A., Beman J.M., Abell G., Philippot L., Prosser J. (2016). Microbes as engines of ecosystem function: When does community structure enhance predictions of ecosystem processes?. Front. Microbiol..

[8] Pimentão A.R., Pascoal C., Castro B.B., Cássio F. (2020). Fungistatic effect of agrochemical and pharmaceutical fungicides on non-target aquatic decomposers does not translate into decreased fungi- or invertebrate-mediated decomposition. Sci. Total Environ..

[9] Huber A., Bach M., Frede H.G. (2000). Pollution of surface waters with pesticides in Germany: Modeling non-point source inputs. Agriculture. Ecosyst. Environ..

[10] Schulz R. (2004). Field studies on exposure, effects, and risk mitigation of aquatic nonpoint-source insecticide pollution—A review. J. Environ. Qual..

[11] Rasmussen J.J., Wiberg-Larsen P., Baattrup-Pedersen A., Monverg R.J., Kronvang B. (2012). Impacts of pesticides and natural stressors on leaf litter decomposition in agricultural streams. Sci. Total Environ..

[12] Helander M., Pauna A., Saikkonen K., Saloniemi I. (2019). Glyphosate residues in soil affect crop plant germination and growth. Sci. Rep..

[13] Leino L., Tall T., Helander M., Saloniemi I., Saikkonen K., Ruuskanen S., Puigbò P. (2021). Classification of the glyphosate target enzyme (5-enolpyruvylshikimate-3-phosphate synthase) for assessing sensitivity of organisms to the herbicide. J. Hazard. Mater..

[14] Peruzzo P.J., Porta A.A., Ronco A.E. (2008). Levels of glyphosate in surface waters, sediments and soils associated with direct sowing soybean cultivation in north pampasic region of Argentina. Environ. Pollut..

[15] Giesy J.P., Dobson S., Solomon K.R. (2000). Ecotoxicological risk assessment for roundup herbicide. Reviews of Environmental Contamination and Toxicology.

[16] Guilherme S., Santos G.M.A., Pacheco M. (2010). European eel (*Angilla anguilla*) genotoxic and pro-oxidant responses following short-term exposure to Roundup—A glyphosate-based herbicide. Mutagenesis.

[17] Glozier N.E., Struger J., Cessna A.J., Gledhill M., Rondeau M., Ernst W.R., Sekela M.A., Cagampan S.J., Sverko E., Murphy C. (2012). Occurrence of glyphosate and acidic herbicides in select urban rivers and streams in Canada. Environ. Sci. Pollut. Res. Int..

[18] Harayashiki C.A.Y., Varela A.S., de Souza Machado A.A., da Costa Cabrera L., Primel E.G., Bianchini A., Corcini C.D. (2013). Toxic effects of the herbicide Roundup in the guppy *Poecilia vivipara* acclimated to fresh water. Aquat. Toxicol..

[19] Schrauzer G.N. (2002). Lithium: Occurrence, Dietary Intakes, Nutritional Essentiality. J. Am. Coll. Nutr..

[20] Tanveer M., Hasanuzzama M., Wang L. (2019). Lithium in environment and potential targets to reduce lithium toxicity in plants. J. Plant Growth Regul..

[21] Leal V.M., Ribeiro J.S., Coelho E.L.D., Freitas M.B.J.G. (2023). Recycling of spent lithium-ion batteries as a sustainable solution to obtain raw materials for different applications. J. Energy Chem..

[22] Shahzad B., Ughal M.N., Tanveer M., Gupta D., Abbas G. (2017). Is lithium biologically an important or toxic element to living organisms? An overview. Environ. Sci. Pollut. Res. Int..

[23] Brown E.E., Gerretsen P., Pollock B., Graff-Guerrero A. (2018). Psychiatry benefits of lithium in water supplies may be due to protection from the neurotoxicity of lead exposure. Med. Hypotheses.

[24] Kavanagh L., Keohane J., Cleary J., Cabellos G.G., Lloyd A. (2017). Lithium in the natural waters of the South East of Ireland. Int. J. Environ. Res. Public Health.

[25] Liu D., Gao L., Zhang Z., Tao S., Pang Q., Li A., Deng H., Yu H. (2018). Lithium promotes the production of reactive oxygen species via GSK-3b/TSC2/TOR signaling in the gill of zebrafish (*Danio rerio*). Chemosphere.

[26] Ewuzie U., Nnorom I.C., Eze S.O. (2020). Lithium in drinking water sources in rural and urban communities in Southeastern Nigeria. Chemosphere.

[27] Sobolev O.I., Gutyj B.V., Darmohray L.M., Sobolievа S.V., Ivanina V.V., Kuzmenko O.A., Karkach P.M., Fesenko V.F., Bilkevych V.V., Mashkin Y.O. (2019). Lithium in the natural environment and its migration in the trophic. Ukr. J. Ecol..

[28] Barbosa H., Soares A.M.V.M., Pereira E., Freitas R. (2023). Lithium: A review on concentrations and impacts in marine and coastal systems. Sci. Total Environ..

[29] Muturi E.J., Donthu R.K., Fields C.J., Moise I.K., Kim C.-H. (2017). Effects of pesticides on microbial communities in container aquatic habitats. Sci. Rep..

[30] Gessner M.O., Graça M.A.S., Barlocher F., Gessner M.O. (2005). Ergosterol as a measure of fungal biomass. Methods in Study Litter Decomposition: A Practical Guide.

[31] Feckler A., Bundschuh M. (2020). Decoupled structure and function of leaf-associated microorganisms under anthropogenic pressure: Potential hurdles for environmental monitoring. Freshw. Sci..

[32] Fernandes I., Pascoal C., Cássio F. (2011). Intraspecific traits change effects on ecosystem functioning under metal stress. Oecologia.

[33] Duarte S., Cássio F., Pascoal C., Barlocher F. (2017). Taxa-area relationship of aquatic fungi on deciduous leaves. PLoS ONE.

[34] Obojska A., Lejczak B., Kubrak M. (1999). Degradation of phosphonates by Streptomycetes isolates. Appl. Microbiol. Biotechnol..

[35] Ternan N.G., Mc Grath J.W., McMullan G., Quin J.P. (1998). Review: Organophosphonates: Occurrence, synthesis and biodegradation by microorganisms. World J. Microbiol. Biotechnol..

[36] Bier R.L., Bernhardt E.S., Boot C.M., Graham E.B., Hall E.K., Lennon J.T., Nemergut D.R., Osborne B.B., Ruíz-González C., Schimel J.P. (2015). Linking microbial community structure ad microbial processes: An empirical and conceptual overview. FEMS Microbiol. Ecol..

[37] Pascoal C., Cássio F., Marvanová L. (2005). Anthropogenic stress may affect aquatic hyphomycetes diversity more than leaf decomposition in a low-order stream. Arch. Hydrobiol..

